# Endovascular approach in the management of idiopathic myointimal hyperplasia of the inferior mesenteric vein

**DOI:** 10.1186/s42155-021-00272-0

**Published:** 2021-12-18

**Authors:** Yash B. Shah, Derek Lee, Tamim S. Khaddash

**Affiliations:** 1 Sidney Kimmel Medical College, Thomas Jefferson University, 1025 Walnut St, 19107 Philadelphia, PA USA; 2 Department of Interventional Radiology, Hospital of the University of Pennsylvania, 3400 Spruce St, 19104 Philadelphia, PA USA

**Keywords:** Embolization, Mesenteric fistula, IMHMV, Ischemic colitis, Arteriovenous malformations, Endovascular treatment

## Abstract

**Background:**

Idiopathic myointimal hyperplasia of the mesenteric vein (IMHMV) is a rare, often undiagnosed pathology affecting the colon. Patients typically present with severe abdominal pain and inflammation caused by smooth muscle proliferation of the veins, leading to arterialization, stenosis, and potential occlusion. The etiology remains unclear, but it has been hypothesized that an arteriovenous connection may be associated with the pathology. This is the first reported case indicating such an association. This case additionally highlights the potential utility of endovascular treatment, as endovascular embolization is generally a less invasive alternative to surgical resection in the treatment of such vascular disorders.

**Case Presentation:**

This report describes a 24-year-old female patient with findings of colitis and an abnormal arteriovenous connection of the inferior mesenteric arterial and venous systems. Partial embolization of this arteriovenous connection temporarily improved the patient’s condition, but her symptoms ultimately returned due to the presence of multiple smaller feeder vessels not amenable to embolization, necessitating colonic resection for definitive treatment. Although prior reports have hypothesized that arterial pressurization of the veins may precipitate myointimal hyperplasia, to the authors’ knowledge, this is the first report of IMHMV with an associated abnormal arteriovenous connection.

**Conclusions:**

This case illustrates the possibility of an association between an arteriovenous connection and IMHMV. This rare diagnosis should be considered in patients with a similar presentation of abdominal pain after common etiologies like IBD have been excluded.

## Background

Idiopathic myointimal hyperplasia of the inferior mesenteric vein (IMHMV) is a rare disorder with only 34 documented cases in the literature. IMHMV typically affects the distal and rectosigmoid colon, and its clinical presentation and pathogenesis resemble that of inflammatory bowel disease (IBD) (Kelly Wu et al. 2020). However, biopsy of the affected colon may show findings of ischemia without classic IBD features (Yantiss et al. 2017). Prevalence is likely underreported, as the disease typically remains undiagnosed during the clinical course until histopathology of resected colon is reviewed. The etiology and pathogenesis of IMHMV are unclear.

This report describes the unique clinical progression of a patient with clinical and radiologic findings of colitis in addition to an abnormal arteriovenous connection of the inferior mesenteric artery (IMA) and the venous system. Prior reports have hypothesized that arterial pressurization of the venous vasculature may cause venous myointimal hyperplasia, but to the authors’ knowledge, there have been no reports of IMHMV in a patient with angiographic evidence of an abnormal arteriovenous connection.

Partial endovascular embolization of this arteriovenous connection yielded temporary improvement of the patient’s symptoms, but definitive treatment with partial colonic resection was ultimately required. Further investigation into an association between IMHMV and vascular anomalies like arteriovenous connections may elucidate the utility of endovascular approaches in the diagnostic evaluation and treatment of patients with IMHMV.

## Case Presentation

**Figure 1:**
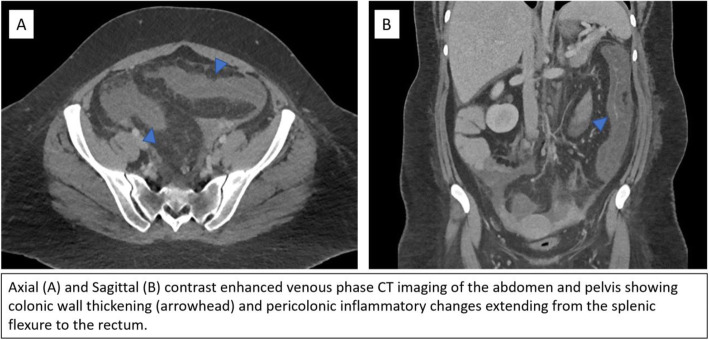
Fig. 1

A 24-year-old female with no significant past medical history initially presented to an outside hospital with severe abdominal pain and scantly bloody stools. She was transferred to a tertiary care center for further work-up and management. Contrast-enhanced CT of the abdomen and pelvis showed findings of colitis extending from the splenic flexure to the rectum (Fig. 1). The patient was empirically treated for infectious and inflammatory etiologies of colitis without improvement. Flexible sigmoidoscopy demonstrated inflammatory changes in the descending colon and rectum with biopsy samples suggestive of ischemic colitis. A small external hemorrhoid was noted. Differential diagnosis for possible ischemic colitis included thrombosis and vasculitis, but there were no definitive imaging findings of either on CT imaging. Hematologic and rheumatologic work-up were nonspecific but notable for elevated rheumatoid factor and positive antinuclear antibodies.

**Figure 2:**
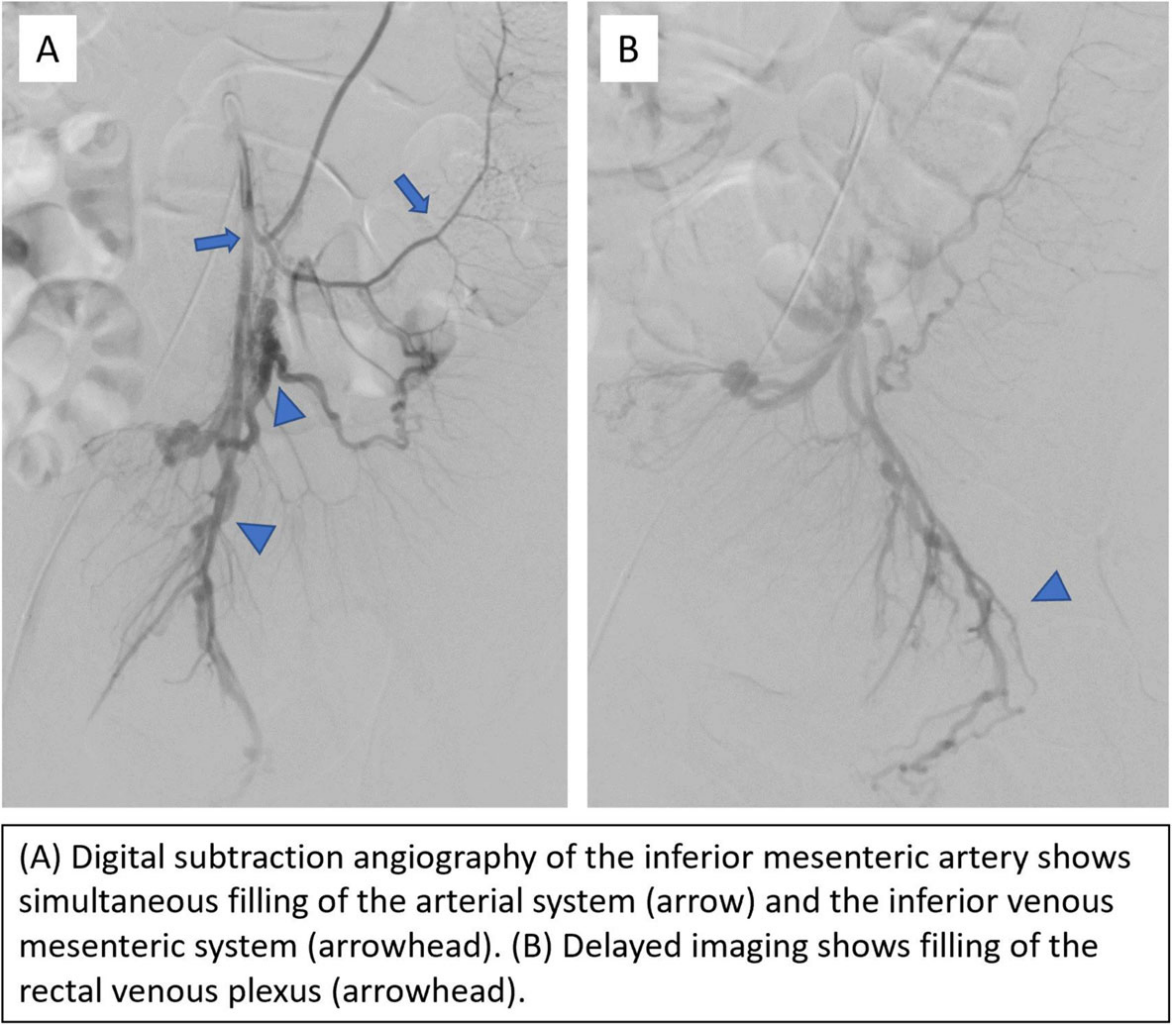
Fig. 2

Interventional radiology was consulted for further angiographic evaluation of the mesenteric vessels given the ischemic findings on pathology. IMA angiography was performed, and prompt filling of the IMA and paralleling mesenteric venous branches was observed (Fig. 2). A few abnormal-appearing, tortuous distal IMA branches were noted. Hepatofugal flow was observed in the IMV.

Given these findings, the patient was empirically treated with steroids and anticoagulation. She showed marginal improvement and had persistent severe abdominal pain. Based on the prompt venous filling seen on conventional angiography, it was hypothesized that the presence of an arteriovenous connection may be causing mesenteric venous congestion. After a multidisciplinary team discussion, it was decided to pursue further angiographic investigation and attempt endovascular embolization of a suspected arteriovenous connection.

**Figure 3:**
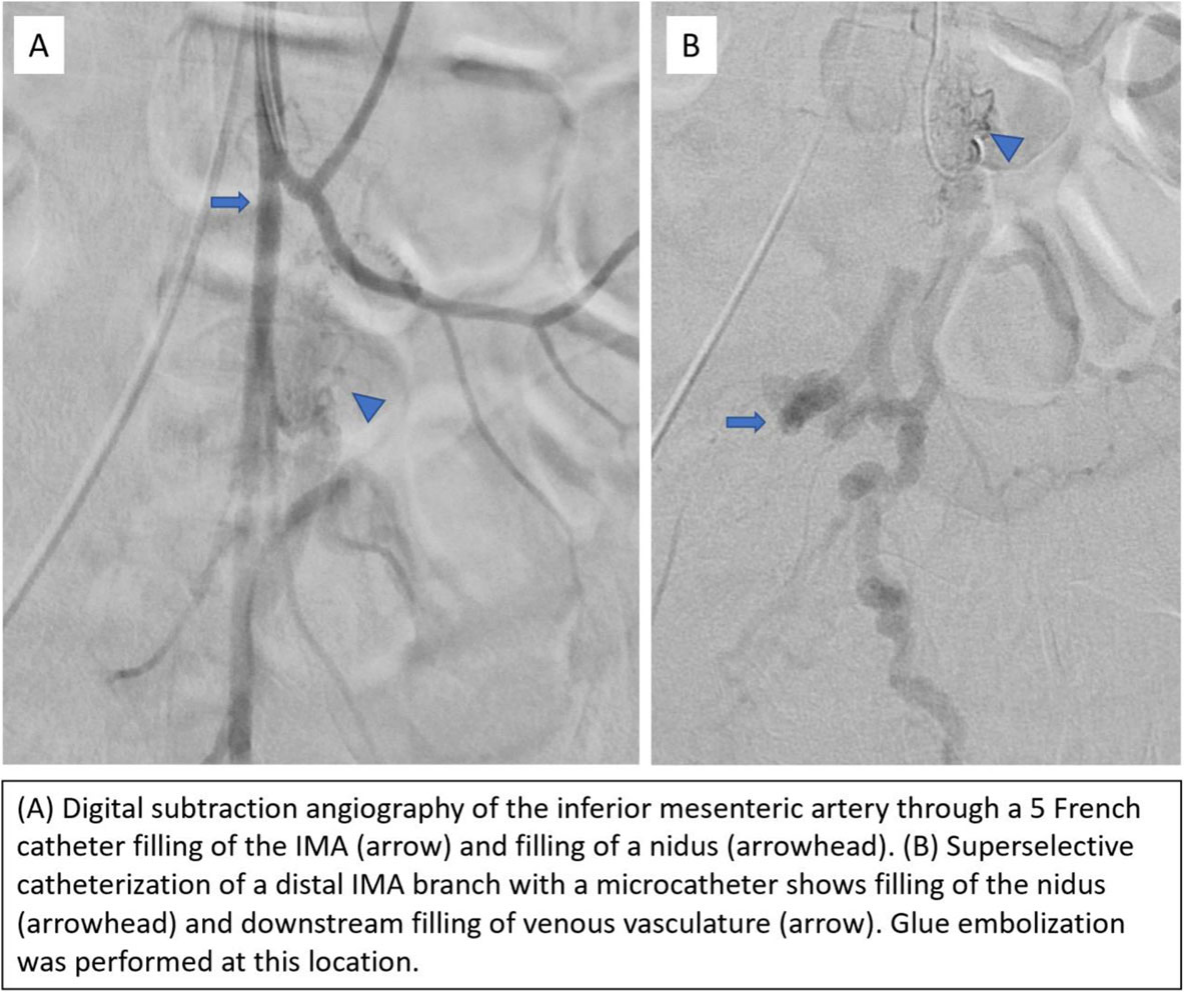
Fig. 3

**Figure 4:**
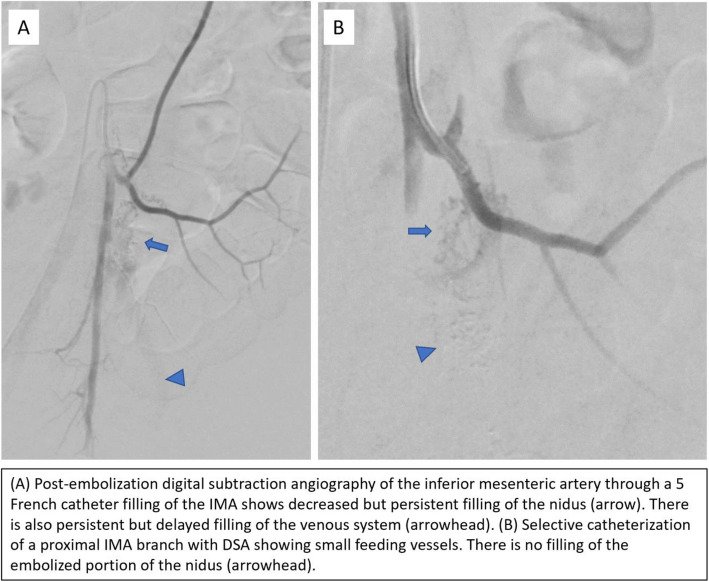
Fig. 4

The patient returned to interventional radiology for further diagnostic imaging and potential intervention. The right common femoral artery was accessed using standard micropuncture technique with a 5 French micropuncture set (Merit Medical, South Jordan, UT). Digital subtraction angiography of the IMA was performed using a 5 French catheter. Using a microcatheter, further selective angiography of the IMA branches was performed. Filling of the venous system was again seen along with a tangle of small vessels compatible with a nidus connecting small arterial feeders to the venous system (Fig. 3). Glue embolization of this nidus was performed using n-butyl cyanoacrylate (n-BCA) (TruFill, Codman and Shurtleff Inc., Raynham, MA) diluted in a 3:1 lipiodol to n-BCA concentration. Post-embolization imaging demonstrated decreased venous filling, although there was persistent venous filling from several smaller IMA feeding branches, which were small and not amenable to embolization (Fig. 4).

The patient’s pain significantly improved the day after embolization, and she was discharged 5 days post-embolization. Unfortunately, her pain returned in 11 days and rose to pre-embolization levels approximately 26 days post-embolization. She was readmitted for pain management, and her deteriorating condition resulted in a multidisciplinary medicine, interventional radiology, and surgery team decision to proceed with colonic resection. Extended left colectomy with partial proctectomy, end transverse colostomy, and rectal stump was performed. The patient tolerated the procedure well, and her pain completely resolved. Pathological analysis of resection specimens was consistent with venous insufficiency, as it indicated prominent concentric intimal smooth muscle hyperplasia with colonic perforation. Dilated capillaries, thickened hyaline walls, and ischemic changes were visualized without evidence of malignancy or thrombosis. Van Gieson’s stain along with venous presence of lymphocytic phlebitis, fat necrosis, and organizing thrombi confirmed IMHMV diagnosis (Genta and Haggitt 1991).

## Conclusions

IMHMV has classically been reported in otherwise healthy young to middle-aged men, although recent cases, including this one, have identified the disease in females or older patients (Platz 2012). Clinical diagnosis of IMHMV is difficult and generally ascertained via histopathology after surgical resection (Platz 2012; Yun et al. 2016). Patients present with nonspecific symptoms that mimic IBD, including severe abdominal pain, weight loss, rectal bleeding, and diarrhea or constipation (Song and Shroff 2017). Patients may also present with subtle bowel perforations (Almumtin et al. 2021). Initial radiologic evaluation of IMHMV patients may yield findings suggestive of colitis, and endoscopy can produce nonspecific inflammatory findings, both of which were seen in this patient.

Patients typically undergo a protracted clinical course of colitis that ultimately requires surgical resection. This patient is unique as her colitis was thought to be secondary to an abnormal arteriovenous connection, which was discovered on conventional angiography performed during mesenteric ischemia work-up.

While uncommon, mesenteric arteriovenous anomalies are well-documented, with multiple case reports describing successful endovascular embolization of arteriovenous fistulas and malformations (Hendy et al. 2018; Hussein 2013; Athanasiou et al. 2014). Endovascular embolization is less invasive and presents fewer complications in comparison to surgical resection, although it does carry potential for bowel ischemia (Hussein 2013). Since only partial embolization was achieved in this case, the interventional radiology team acknowledged the possibility that pain would return as new arterial feeders develop post-embolization and repressurize the venous system. The patient did see significant pain reduction, and her opioid requirement decreased in the subsequent days, but this relief was transient, lasting only 11 days. This certainly raises the possibility of a placebo effect from embolization or temporary improvement from perioperative pain killers. Additionally, it is possible this patient’s abnormal arteriovenous connection may not have represented a true primary arteriovenous malformation (AVM), but a secondary, nonspecific arteriovenous connection associated with either IMHMV and/or chronic thrombosis of the IMV.

The definitive etiologies of both mesenteric AVM and IMHMV are unknown. It is plausible that AVM and IMHMV are associated, but no previous studies have proven the basis of this association. IMHMV is characterized by smooth muscle proliferation in small and medium-sized mesenteric veins, in essence creating venous arterialization that leads to stenosis and possible occlusion. Thus, prior reports have hypothesized that IMHMV results from an arteriovenous connection that leads to increased pressurization of the veins, resulting in venous arterialization.

Standard treatment for both IMHMV and AVM is surgical resection of the affected section of bowel. The median time from patient presentation and symptom onset to surgical resection is 5 months. However, endovascular embolization has also been shown to be a viable treatment option for arteriovenous connections where the anatomy is amenable. There have not yet been any reports of successful conservative medical treatment for IMHMV. Typically, the diagnosis is missed, and surgery is performed after significant morbidity or failure to respond to IBD medical treatments (Bronswijk et al. 2019).

This case illustrates the possible significance of an arteriovenous connection in the setting of ischemic colitis with a plausible pathogenic relationship with IMHMV. Coupling this case with the previously hypothesized associations with vascular anomalies, early radiologic evaluation for an abnormal arteriovenous connection may facilitate a diagnosis of IMHMV. While embolization did not yield long-term benefit in the presented case, further study is needed to investigate the utility of endovascular approaches as a viable treatment alternative to resection for IMHMV with an abnormal arteriovenous connection, particularly when diagnosed early, as endovascular treatment offers significant benefits (Hendy et al. 2018). Earlier IMHMV identification may also yield earlier diagnosis and improved outcomes for patients with neuroendocrine tumors, which have been shown to be associated with this condition (Guadagno et al. 2016). With increased awareness of the condition, IMHMV can be considered in the differential diagnoses of patients presenting with protracted severe abdominal pain and colitis without a clear infectious or inflammatory etiology.

## Data Availability

Data sharing is not applicable to this article as no datasets were generated or analyzed during the current study.

## Abbreviations

IMHMV: idiopathic myointimal hyperplasia of the mesenteric vein
IBD: inflammatory bowel disease
IMA: inferior mesenteric artery
AVM: arteriovenous malformation
